# A facile non-solvent induced phase separation process for preparation of highly porous polybenzimidazole separator for lithium metal battery application

**DOI:** 10.1038/s41598-019-55865-6

**Published:** 2019-12-17

**Authors:** Jiaying Wang, Yang He, Quan Wu, Yunfeng Zhang, Zhiyuan Li, Zhihong Liu, Shikang Huo, Jiaming Dong, Danli Zeng, Hansong Cheng

**Affiliations:** 1 Sustainable Energy Laboratory, Faculty of Material Science and Chemistry, China University of Geosciences (Wuhan), 388 Lumo RD, Wuhan, 430074 China; 2 School of Environment and Civil Engineering, Dongguan University of Technology, No.1, Daxue Road, Songshan Lake, Dongguan, Guangdong Province 523808 P. R. China; 3 National Quality Supervision & Inspection Center of Lithium Battery Products (Shandong), Intelligent Manufacturing Town, Fuyuan 3rd Road, National High-tech Zone, Zaozhuang, Shandong 277800 P. R. China

**Keywords:** Energy, Energy science and technology

## Abstract

The drawbacks of low porosity, inferior electrolyte wettability, low thermal dimensional stability and permissive lithium dendrite growth of the conventional microporous polyolefin-based separators hinder their widely application in the high power density and safe Lithium ion batteries. Herein, highly porous polybenzimidazole-based separator is prepared by a facile non-solvent induced phase separation process (NIPS) using water, ethanol, chloroform and ethyl acetate as the coagulation bath solvent, respectively. It was found that the ethanol is suitable to fabricate uniform morphology macroporous separator with the porosity of 92%, electrolyte uptake of 594 wt.%, and strong mechanical strength of 15.9 MPa. In addition, the experimental tests (electrochemical analysis and XPS test) and density functional theory calculation suggest that the electron-rich imidazole ring of polybenzimidazle can enhance Li^+^ mobility electrostatic attraction interaction while the block the PF_6_^−^ mobility via electrostatic repulsion interaction. Therefore, high Li^+^ transference number of 0.76 was obtained for the neat polybenzimidazole-based polymer electrolyte. As a proof of concept, the Li/LiFePO_4_ cell with the polybenzimidazole-based polymer electrolyte/1.0 M LiPF_6_^−^ ethylene carbonate/dimethyl carbonate (v:v = 1:1) electrolyte exhibits excellent rate capability of >100 mAh g^−1^ at 6 C (1 C = 170 mA g^−1^) and superior cycle stability of 1000 cycles.

## Introduction

Anode materials with higher specific capacity than the commercialized graphite are currently one of the most challenges for the next-generation battery systems^1^. Among them, Li metal anode has attracted much attention in the past decade due to its highest specific capacity (3860 mAh g^−1^) and the lowest potential (−3.04 V vs. NHE)^2^. However, the uncontrolled dendritic and mossy lithium growth leads to short cycle life, low coulombic efficiency and even serious safety issues by the internal short circuit^3^. Numerous efforts have been devoted to increasing Li^+^ transference number of electrolyte for uniform deposition of lithium to suppress its dendrite growth^4–6^. Recently, single ion conducting polymer electrolytes with Li^+^ transference number close to 1.0 have been proved as an effective strategy^7–12^. Unfortunately, the high cost of both materials and film-forming process largely hinder their commercialization. Another approach to increase Li^+^ transference number is to add a promoter and enhance the Li^+^ dissociation, such as metal oxides^13^, ceramics^14,15^ or MOFs^16^. However, the precipitation of promoters from the membrane will lead to irreversible performance loss^17,18^.

The high thermal dimensional stability and fire retardancy of separators are of great importance to mitigate fire hazards by avoiding the short circuit^19,20^. The commercial microporous polyolefin separators, i.e., polyethylene (PE) and polypropylene (PP), suffer from severe thermal dimensional shrinkage at elevated temperatures (100–150 °C)^21^. In addition, the low porosity (30–40%) largely limits the electrolyte uptake, leading to low ionic conductivity^22–24^. Moreover, the polyolefin based separators have poor compatibility with the electrolyte due to its hydrophobicity, which hampered fast absorption of large amount of electrolytes for effective ionic mobility^25^.

As one of the most popular engineering plastics, the fully aromatic polybenzimidazole (PBI) has intrinsically high thermal stability (thermal decomposition, T_d_ > 550 °C). Li *et al*. fabricated the porous PBI-based separator via water vapor induced phase immersion method, the as-prepared membrane possesses the porosity of 81%, and electrolyte uptake of 328%^26^. Liang and his co-workers performed a blending phase inversion to prepare the micropores of PBI-based membrane via extracting poly(ethylene glycol)−10000 (PEG-10000) from the dry OPBI/PEG blend membranes with water^27^. The high porosity of 70% and electrolyte uptake of 316% were achieved when the PEG-10000 content was up to 150 wt.%. However, the mechanical property was largely sacrificed and the mechanical strength was decreased to 10 MPa, which is about two-thirds of commercial Celgard 2400 separator.

The lithium ion transference number (${t}_{Li}^{+}$) is one of the most critical parameters of the electrolyte for application in lithium metal batteries by suppressing lithium dendrite growth. The relationship between the ${t}_{Li}^{+}$ and lithium dendrite growth can be illustrated by using the Equation: ${\rm{\tau }}={\rm{\pi }}{\rm{D}}(\frac{e{C}_{0}}{2J{t}_{-}})$, where τ is time consume to achieve complete ion depletion, *D* is the ambipolar diffusion coefficient, *e* is the electric charge, *C*_0_ is the initial concentration, *J* is the current density and *t*_*-*_ is the transference number of anions. It indicates that reduce the t_−_ or increase transference of ${t}_{Li}^{+}$ can increase the deplete time of Li^+^ and suppress lithium dendrite growth.

**Figure 1:**
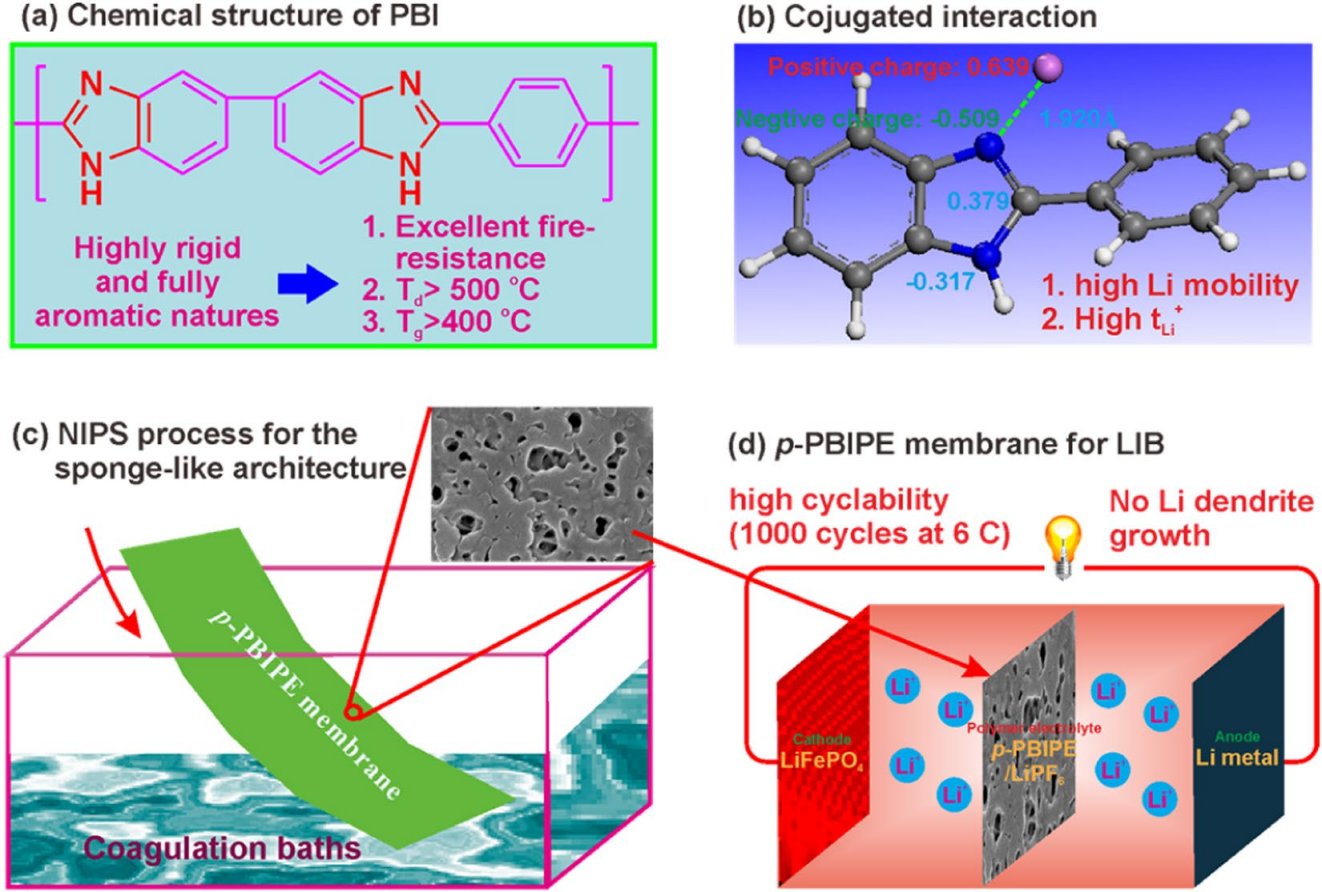
(**a**) Schematic illustration of the composition and advances of PBI, (**b**) conjugated interaction the PBI and Li^+^, (**c**) fabrication of *p*-PPBIPE membrane, and (**d**) Li/LiFePO_4_ cell with *p*-PPBIPE membrane.

In the present, a facile non-solvent induced phase separation process was performed to fabricate highly porous PBI-based membranes by using four different non-solvent, water, ethanol, chloroform and ethyl acetate as the coagulation bath solvent, respectively (Fig. 1b). The results indicate that ethanol induced phase separated porous membrane possesses highest porosity of 92% and electrolyte uptake of 594%, which are almost 3 times and 7 times higher than those of commercial PP separator, respectively. In addition, the porous membrane displays a strong mechanical strength of 15.8 MPa, which is even higher than the value of 14.5 MPa for commercial PP separator. It is beneficial for both incorporation of more electrolytes and uniform Li stripping/plating^28,29^. In addition, the strong electron-rich feature of imidazole ring favors Li^+^ dissociation from lithium salt by the strong conjugated interaction (Fig. 1c), enhances Li^+^ diffusion and hence its transference number. The Li^+^ transference number of PBI-based polymer electrolytes without any additives is as high as 0.76^30^. After impregnation of 1.0 M LiPF_6_^-^ EC/DMC (v:v = 1:1) into the *p*-PBIPE membrane, the Li/LiFePO_4_ cell (Fig. 1d) exhibits excellent rate capability and superior cycle stability for over 1000 cycles at 6 C. These make the PBI-based polymer electrolyte for advanced Li metal batteries with high safety and performance.

## Results and Discussion

### Membrane morphology

**Figure 2:**
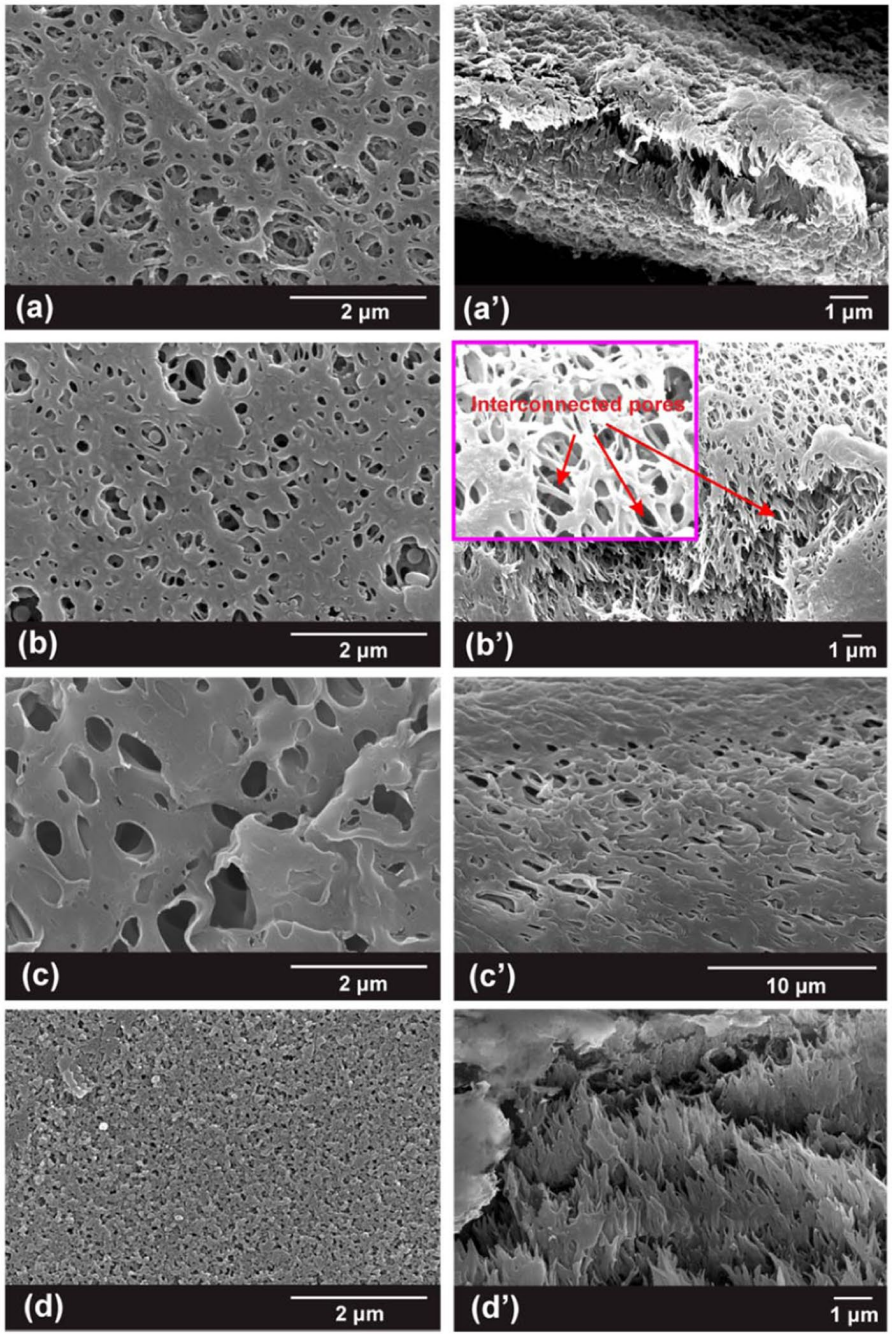
SEM images of as-prepared *p*-PBIPE membranes. Surfaces: (**a**) *wp*-PBIPE, (**b**) *ep*-PBIPE, (**c**) *ap*-PBIPE, and (**d**) *cp*-PBIPE; cross-section: (a’) *wp*-PBIPE, (b’) *ep*-PBIPE, (c’) *ap*-PBIPE, and (d’) *cp*-PBIPE.

To prepare a polymer electrolyte membrane with high porosity and appropriate mechanical strength, four solvents (water, ethanol, ethyl acetate and chloroform) are selected as the coagulation bath for the NIPS process, where the as-prepared membranes are denoted as *wp*-PBIPE, *ep*-PBIPE, *ap*-PBIPE, and *cp*-PBIPE, respectively. Scanning electron microscopy (SEM) images of the four membranes are shown in Fig. 2, and the key parameters, such as porosity, electrolyte uptake and mechanical strength, are listed in Table 1. The *wp*-PBIPE (Fig. 2a and a’) and *ep*-PBIPE (Fig. 2b and b’) membranes display more favorable pore architectures than that of the *ap*-PBIPE (Fig. 2c and c’) and *cp*-PBIPE (Fig. 2d and d’) membranes. Simultaneously, the *wp*-PBIPE and *ep*-PBIPE show much higher porosities and more electrolyte uptake than those of the *ap*-PBIPE and *cp*-PBIPE (Table 1). The *ap*-PBIPE exhibits the highest mechanical strength of 23.5 MPa, while the high toxicity as well as high cost of the ethyl acetate hinders its large-scale application. In addition, the non-uniform pore architecture gives rise to detrimental effect on the battery performances^31^. In particular, the *ep*-PBIPE exhibits high porosity and appropriate mechanical strength of 15.9 MPa, higher than mechanical strength of 14.5 MPa in the transverse direction for the commercial Celgard separator^32,33^, which is greatly beneficial for improving battery cycle stability^34,35^. Furthermore, the *ep*-PBIPE displays uniform and interconnected pore structure, indicating the smooth Li^+^ diffusion, which is similar to the commercial Celgard separator (Fig. S1). Besides, the low cost and low toxicity of the ethanol coagulation bath makes the *ep*-PBIPE promising for large-scale application.

**Table 1:** Tabulated properties of the porosity, electrolyte uptake, and mechanical strength of the as-prepared *p*-PBIPE membranes.

Properties	Thickness (μm)	Porosity(%)	Electrolyte uptake (%)	Mechanical strength (MPa)	Elongation (%)
*wp*-PBIPE	29	87	614	2.3	4.5
*ep*-PBIPE	33	92	594	15.9	7.6
*ap*-PBIPE	31	85	521	23.5	13.0
*cp*-PBIPE	34	82	507	8.4	3.1
Celgard separator	23	31	80	14.5	824.9

### Conjugated interaction

**Figure 3:**
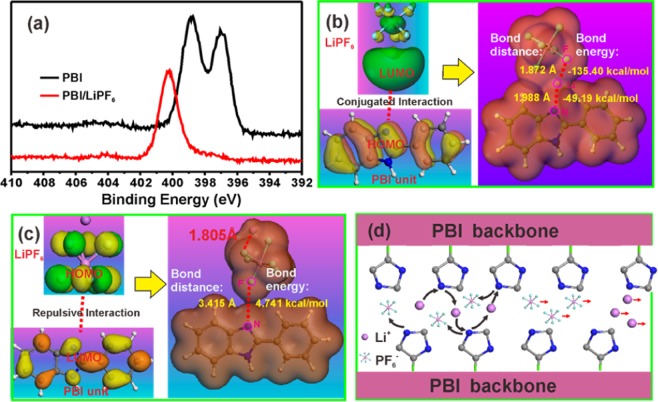
(**a**) XPS spectra of N1s of the neat PBI membrane and the PBI immersed in 1.0 M LiPF_6_^-^EC/DMC (v:v = 1:1); (**b**) The calculated electrostatic potential of LiPF_6_, PBI unit and PBI unit/LiPF_6_ to illustrate the conjugated interaction between Li^+^ and electron-rich imidazole ring; (**c**) The repulsive interaction between PF_6_^−^ and electron-rich imidazole ring; (**d**) Schematic illustration of the electron-rich imidazole ring for promoting the Li^+^ mobile and restricting PF_6_^-^ mobile.

The strong interaction between Li^+^ and electron-rich imidazole ring to promote Li^+^ diffusion are proved by X-ray photoelectron spectroscopy (XPS) and density functional theory (DFT) calculations. As depicted in Fig. 3a, two peaks are observed at around 398.7 eV and 397.1 eV for the neat PBI membrane, corresponding to the –N = (*sp*^2^ hybrid N atom) and –NH– (*sp*^3^ hybrid N atom) of the imidazole ring, respectively. However, one peak disappears after immersing the PBI membrane into 1.0 M LiPF_6_ in EC/DMC (v:v = 1:1) electrolyte and another peak shifts to 400.3 eV, indicating that the strong interaction forms between LiPF_6_ and PBI backbone.

As illustrated in Fig. 3b and Table S1, DFT calculations indicate that the highest occupied molecular orbital (HOMO) of the electron-rich imidazole ring in PBI can matches well with the lowest unoccupied molecular orbital (LUMO) of the Li^+^ in LiPF_6_ to form N–Li conjugated bond with the bonding distance of 1.988 Å and bonding energy of −49.194 kcal mol^−1^. By contrast, the electron-rich imidazole ring shows repulsive interaction to PF_6_^-^ with long bonding distance of 3.415 Å and bonding energy of 4.741 kcal mol^−1^ (Figs. 3c and S2). The formed conjugated bond contributes to the Li^+^ dissociation from PF_6_^−^, serving as another Li^+^ pathway in *ep*-PBIPE membrane while the movement of PF_6_^-^ is restricted (Fig. 3d).

### Li^+^ transference number, Li stripping/plating performance and morphology of Li metal anode

**Figure 4:**
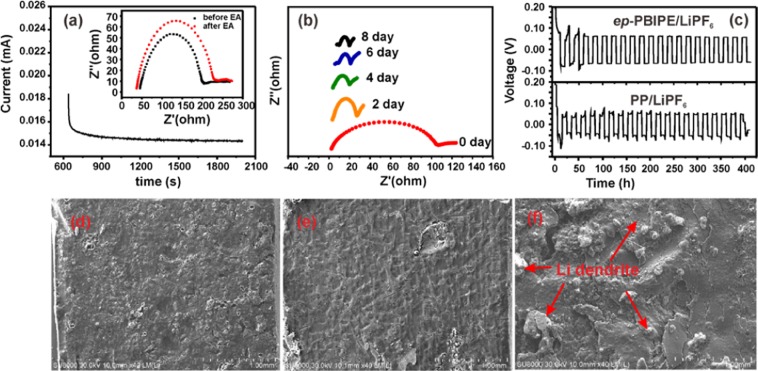
(**a**) The time-dependence response of dc polarization (inserted impedance spectra) for the *ep*-PBIPE/LiPF_6_^-^EC/DMC electrolyte on the Li|*ep*-PBIPE/LiPF_6_|Li symmetric cell, (**b**) Time evolution of the impedance response of a symmetrical Li|*ep*-PBIPE/LiPF_6_|Li cell (**c**) Galvanostatic cycling of symmetric lithium cells with the *ep*-PBIPE/LiPF_6_^-^ EC/DMC electrolyte and the Celgard/LiPF_6_^-^EC/DMC electrolyte. SEM images of Li foil after long-term stripping/plating with *ep*-PBIPE/LiPF_6_^-^EC/DMC electrolyte (**d**), pristine Li foil (**e**) and Celgard/LiPF_6_^-^EC/DMC electrolyte (**f**).

The Li ion transference number of *ep*-PBIPE/LiPF_6_ was measured by a steady-state current method on the Li|electrolyte|Li symmetric cell asseblied with the *ep*-PBIPE/LiPF_6_ and PP-LiPF_6_^−^EC/DMC electrolyte at room temperature, the impedance spectra and the time-dependence response upon DC polarization are displayed in Fig. 4a. It shows that the Li ion transference number of *ep*-PBIPE/LiPF_6_ electrolyte consequently increases from 0.23 (Fig. S3) for the Celgard/LiPF_6_ electrolyte up to 0.76, which is beneficial to improve the stability of Li stripping/plating, in particular, to suppress lithium dendrite growth^36^. The time-dependent interface stability between the *ep*-PBIPE/LiPF_6_ electrolyte and Li metal based on impedance measurements of a Li|*ep*-PBIPE/LiPF_6_|Li cell is depicted in Fig. 4b. It depicts that the resistance becomes constant after 4 day, demonstrating a stable interface between the Li and *ep*-PBIPE.

The stripping/plating performance of metallic lithium anodes was determined by the galvanostatic Li stripping/plating cycling test and the results are shown in Fig. 4c. Li/Li symmetric cell at 0.5 mA cm^−2^ with the *ep*-PBIPE/LiPF_6_ electrolyte exhibits a stable voltage profile, which is consistant with the previously reported anion-anchoring single ion conducting polymer electrolytes^37–39^. In contrast, Li/Li symmetric cell with the Celgard/LiPF_6_ electrolyte shows fluctuating voltage profile with increasing voltage hysteresis, stemming from the depletion of cations at the Li/electrolyte interface^40,41^. Figure 4d–f shows the SEM images of Li foil with ep-PBIPE/LiPF_6_ electrolyte and Celgard/LiPF_6_ electrolyte after long cycles of stripping/plating. The top surface of Li electrode with *ep*-PBIPE/LiPF_6_ electrolyte is relatively uniform (Fig. 4d), without an overgrowth of dendrites, which is similar with the neat Li foil (Fig. 4e). In contrast, the Li foil in Celgard/LiPF_6_ electrolyte exhibits a mossy, dendritic deposition (Fig. 4f).

### Thermal stability and fire- retardancy

**Figure 5:**
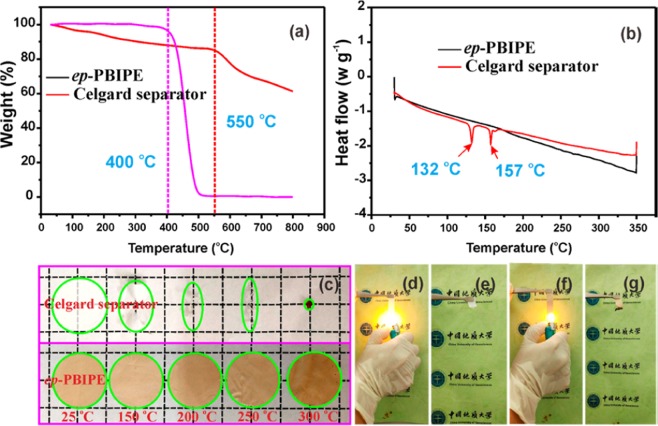
(**a**) TG curves and (**b**) DSC curves of Celgard and *ep*-PBIPE membranes, (**c**) Thermal shrinkage images of Celgard separator and *ep*-PBIPE membrane. Fire-resistance test of Celgard, before (**d**) and after (**e**) burning, and *ep*-PBIPE membrane, before (**f**) and after (**g**) burning.

It is known that good thermal stabilities, including high thermal decomposition temperature and good dimensional stability are key parameters for battery separators, playing a vital role in preventing the thermal runaway when commercial batteries overheat caused by overcharge, overdischarge, internal and external short circuit or accidents^42–44^. Thermo-gravimetric (TG) analysis is employed to investigate the thermal stability of the Celgard separator and *ep*-PBIPE. As shown in Fig. 5a, the thermal decomposition temperatures reach up to 400 °C and 550 °C, respectively, for the Celgard separator and *ep*-PBIPE, which are much higher than the required temperature of 80 °C. However, differential scanning calorimeter (DSC) analysis in Fig. 5b reveals that the Celgard separator possesses soften point and melting point at 132 °C and 157 °C, respectively, representing the poor thermal dimensional stability (Fig. 5c). In contrast, no endothermic peak is observed for the *ep*-PBIPE even at high temperature of 350 °C, which is consistent with the high thermal dimensional stability without any shrinkage at 300 °C. Moreover, the fire-retardancy tests indicate that the *ep*-PBIPE is nonflammable and exhibit intriguing fire-retarding properties, benefiting from the intrinsic flame-retardancy of PBI (Fig. 5d–g).

### Wettability

**Figure 6:**
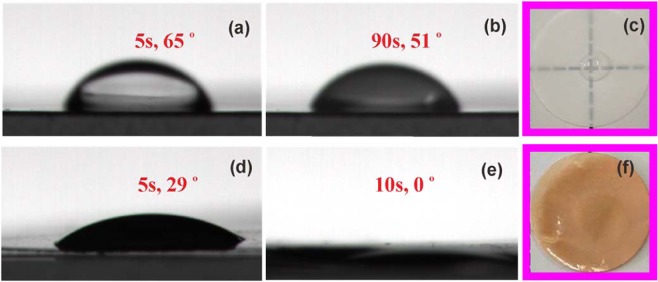
Contact angles of 1.0 M LiPF_6_ in EC/DMC liquid electrolyte on Celgard separator after (**a**) 5 s, (**b**) 90 s, and (**c**) the photo after 60 s, and contact angles of 1.0 M LiPF_6_ in EC/DMC electrolyte on *ep*-PBIPE membrane after (**d**) 5 s, (**e**) 10 s, and (**f**) the image after 60 s wetting.

Figure 6a–f shows the electrolyte wettability of the Celgard separator and *ep*-PBIPE determined by the contact angle measurements. The Celgard separator displays a slow electrolyte wetting with high contact angle of 51° after 90 s (Fig. 6a–c). The poor electrolyte wetting is caused by the nonpolar nature of the polyolefin polymer, originated from an intrinsically hydrophobic property and low surface energy^45,46^. In contrast, the electrolyte completely penetrates into the *ep*-PBIPE within 10 s (Fig. 6d–f). Besides the rather different pore structures, the fast electrolyte wetting of the *ep*-PBIPE is attributed to its highly polar imidazole ring. Accordingly, the superior electrolyte wetting leads to good electrolyte interfacial contact and small interfacial resistance^26,47^.

### Electrochemical performance

**Figure 7:**
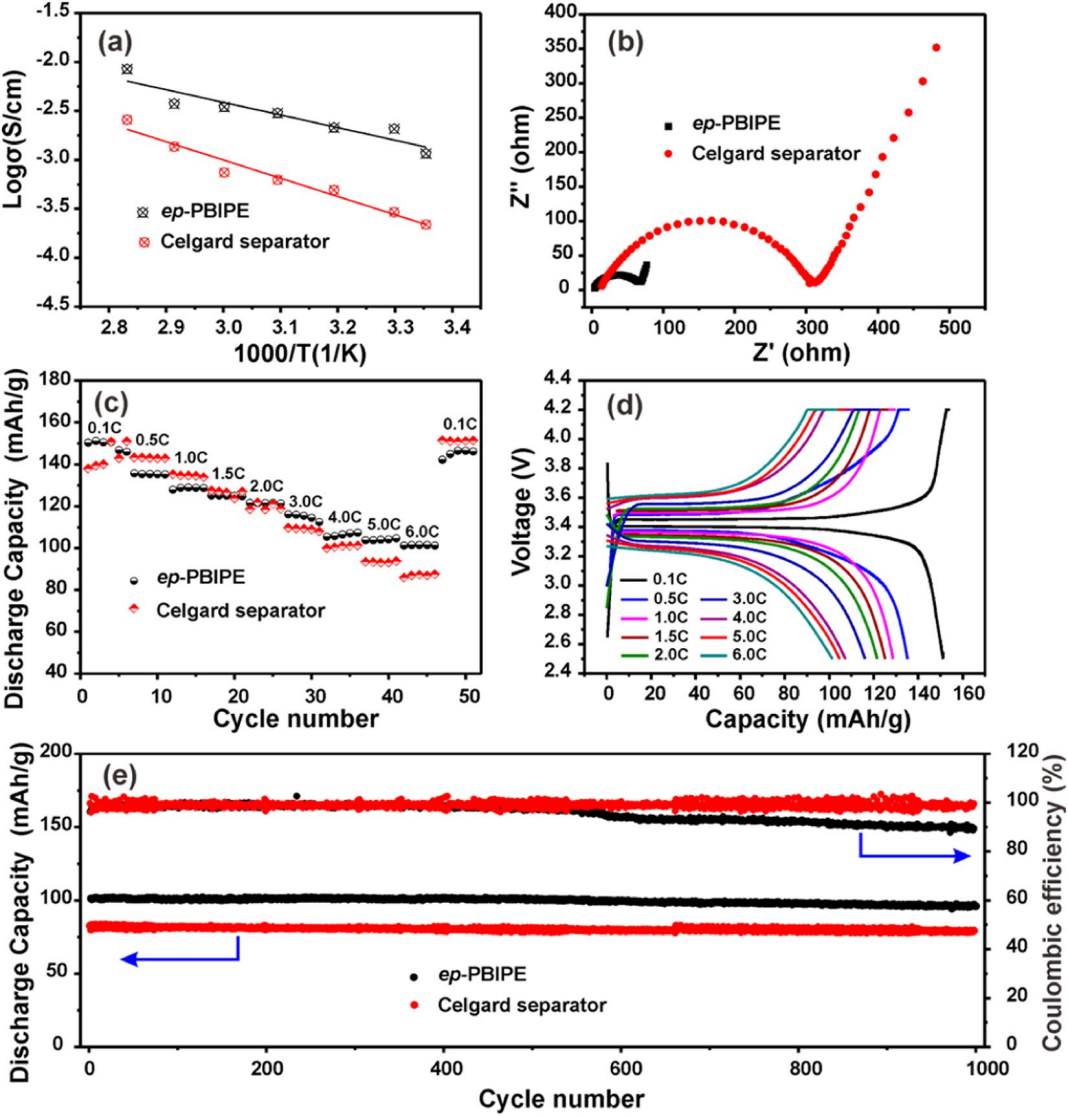
Comparison of electrochemical performances of *ep*-PBIPE and Celgard separator with 1.0 M LiPF_6_ in EC/DMC electrolyte. (**a**) Ionic conductivities; (**b**) Interfacial resistances; (**c**) Rate performances of Li/LiFePO_4_ cells; (**d**) Discharge/charge profiles at selected C-rates; (**e**) Long-term cycle performance at 6 C.

The high ionic conductivity and good electrode compatibility of polymer electrolytes are crucial for the power density of LIBs. The temperature dependence of ionic conductivity of the *ep*-PBIPE membrane and Celgard separator, soaking with 1.0 M LiPF_6_^-^EC/DMC, is depicted in Fig. 7a. The ionic conductivity of the *ep*-PBIPE/1.0 M LiPF_6_^-^EC/DMC is 1.16 mS cm^−1^, which is much higher than that of Celgard/1.0 M LiPF_6_^-^EC/DMC (0.44 mS cm^−1^). Figure 7b shows the Nyquist plots of symmetric cells with two electrolytes. Favored by high porosity to absorb more electrolytes, the assembled cell with *ep*-PBIPE membrane exhibits reduced interfacial resistance.

The electrochemical performances with the *ep*-PBIPE/1.0 M LiPF_6_^-^EC/DMC and Celgard/1.0 M LiPF_6_-EC/DMC electrolytes are evaluated in Li/LiFePO_4_ cells. As depicted in Fig. 7c, Li/LiFePO_4_ cell with the *ep*-PBIPE membrane demonstrates reversible discharge capacity of 151 mAh g^−1^ at 0.1C, close to the theoretical capacity (LiFePO_4_, 170 mAh g^−1^). Even at high current density of 6C, it still maintains high discharge capacity of >100 mAh g^−1^ (Fig. 7d), which is much higher than that of 87 mAh g^−1^ for the cell with Celgard separator. The cycling performances with two electrolytes at 6C are shown in Fig. 7e. The Li/LiFePO_4_ cell with the *ep*-PBIPE-based electrolyte delivers an initial discharge capacity of 101 mAh g^−1^, higher than that of 83 mAh g^−1^ for the Celgard-based electrolyte, and the capacity retention is 92.1% after 1000 cycles, corresponding to a capacity loss of 0.0079% per cycle, which is attributed to excellent electrochemical stability (Fig. S4). Furthermore, we found that the coulombic efficiency of *ep*-PBIPE-based separator is much lower than that of Celgard separator after ~600 cycles. As we know that when the coulombic efficiency reaches below 95%, it suggests the failure of the lithium metal battery. Therefore, the stable cycling might be achieved by overwhelm usage of lithium metal rather than the merits of the separator. These results suggest that the *ep*-PBIPE membrane exhibits high potential application in high-density and safe Li metal batteries.

In conclusion, a series of PBI-based polymer electrolytes (*p*-PBIPEs) were successfully fabricated via a facile non-solvent induced phase separation process. The results indicate that ethanol is the best coagulation bath for highly porous *p*-PBIPE (*ep*-PBIPE) membrane with well-interconnected sponge-like pore structure and appropriate mechanical strength. In particular, the strong conjugated interaction between Li^+^ and electron-rich imidazole ring is greatly favorable for improving the Li^+^ dissociation and migration, subsequently increasing Li^+^ transference number up to 0.76, and can effectively suppress the lithium dendrites growth. This enables stable Li stripping/plating for more than 400 h. In addition, the highly rigid and fully aromatic PBI backbone results in high thermal stability of *ep*-PBIPE membrane even at 300 °C, and the high polarity of imidazole ring and high porosity guarantees fast electrolyte wetting within 10 s. The Li/LiFePO_4_ battery with the *ep*-PBIPE membrane exhibits excellent rate performance of >100 mAh g^−1^ at 6C and outstanding electrochemical stability for 1000 cycles.

## Experimental Section

### Materials

Polybenzimidazoles (PBI) was purchased from Suzhou Pinyu Optoelectronics Technology Co., Ltd. Lithium iron phosphate (LiFePO4), PVDF, and conductive agent (Acetylene Black) were purchased from Shanghai Darui Fine Chemical Co., Ltd., and used as received. N,Ndimethylacetamide (DMAC, AR, Aladdin) and N-Methyl-2-pyrrolidone (NMP, Anhydrous grade, 99.5%, Aladdin) were used as received. PP membrane (Celgard 2400) was cut into 19 mm film for use.

### Fabrication of p-PBIPE membranes

**Figure 8:**
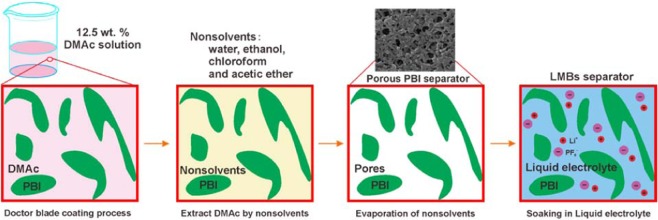
The illustration of the NIPS process for preparation of the porous PBI separator.

The non-solvent induced phase separation process was conducted according to our previous publication^45^. To investigate the effect of different coagulation bath solvents on the properties of the membranes, the four solvents with different polarity, water, ethanol, ethyl acetate and chloroform, were selected as solvents. As illustrated in Fig. 8, the calculated amount of PBI was first dissolved in N, N-dimethylacetamide (DMAc) to prepare a 12.5 wt% uniform casting solution. Afterward, the homogenous solution was cast onto a clean glass plate to prepare a film with average thickness of 35 µm. The film was quickly immersed in the mentioned four coagulation baths for 0.5 h to exchange DMAc solvent. Finally, the formed *p*-PBIPEs were dried at room temperature for 12 h followed by at 100 °C for 24 h in a vacuum oven.

### Preparation of LiFePO_4_ electrode and battery assembly

The LiFePO_4_ cathode consisting of a mixture of LiFePO_4_ active material, acetylene black (AB) and binder of PVDF at a mass ratio of 7:2:1 was prepared by the following steps. PVDF binder was firstly dissolved in NMP, and the well-mixed mixture was then added into the above solution and stirred for at least 4 h to form homogeneous slurry. After that, the slurry was cast on an aluminum foil, dried at 60 °C for 12 h, and cut into 15 mm discs for use as the cathode of a coin cell and the mass loading of the cathode is approximately 4 mg cm^−2^. Finally, the as-prepared cathode film was completely dried at 80 °C in a vacuum oven overnight and then transferred into an argon-filled glove box for further characterization. The Li/LiFePO_4_ half-cells were assembled with *p*-PBIPE membrane and Celgard as separators and 1.0 M LiPF_6_^-^EC/DMC (v/v = 1:1) as electrolyte.

### Characterization

The morphology of the membranes was studied using a scanning electron microscope (FE-SEM, SU8010, HITACHI). Samples must be sprayed with gold for 100 s before testing. The interaction between *p*-PBIPEs membrane and LiFP_6_ was analyzed by the X-ray photoelectron spectroscopy (XPS, Escalab 250xi, Thermo Fisher) and the testing samples were the *p*-PBIPEs membranes before and after immersed in 1 M LiPF_6_/EC/DMC (v:v = 1:1). The tensile strength and elongation tests of membranes were indicated using a tensile tester XLW (PC) at a strain rate of 25 mm/min. The thermal stability of membranes was characterized thermogravimetric analyzer (STA 449 F3, Germany NETZSCH) under an atmosphere of nitrogen with temperature range of from 30 to 800 °C at a rate of 10 °C min^−1^. The endothermic and exothermic behaviors were investigated using Differential Scanning Calorimetry (METTLER TOLEDO DSC3) under nitrogen flow from 30 to 350 °C. The wettability of membranes with electrolyte was conducted by static contact angle and electrolyte spreading test. The thermal shrinkage test was performed by putting the membranes in a heating plate at various temperatures (150, 200, 250 and 300 °C), for 0.5 h at each temperature. The electrochemical stability was measured by the cyclic voltammetry (CV) with the membrane was sandwiched by a lithium electrode and a stainless steel (SS) electrode. The voltage was swept at the scan rate of 0.5 mV s^−1^ from −1.0 V to 5.0 V (vs. Li/Li^+^).

The electrolyte uptake of the membrane was measured by immersing the membrane in a liquid electrolyte (1 M LiPF_6_ in EC / DMC, 1:1 v/v) for 24 h for achieving complete soakage. The weights of dry membrane (M_0_) and wet membrane (M_1_) were obtained by accurately weighing the mass before and after the membrane soakage. The electrolyte uptake was calculated by the following Eq. (1):

1 $${\rm{Solvent}}\,{\rm{uptake}}=\frac{{M}_{1}+{M}_{0}}{{M}_{0}}\times 100 \% $$

The process for determination of porosity (P) of the membrane is as follows. The dry membrane was immersed in n-butanol for 24 h for soaking the solvent in pore. The weight of the dry membrane and wet membrane were measured, respectively, and then the porosity was calculated according to the following Eq. (2):

2 $$P \% =\frac{{W}_{w}-{W}_{d}}{\rho b\,\ast \,{V}_{m}}\times 100 \% $$

where W_d_ is the dry weight of membrane, W_w_ is the wet weight of membrane, ρ_b_ is the density of n-butanol and V_m_ is the volume of the membrane.

The ionic conductivity (σ) of polymer electrolytes was tested using electrochemical impedance spectroscopy (EIS) by sandwiching the electrolyte-soaked membrane between two stainless steel (SS) electrodes. The impedance data was recorded over a frequency range from 10^6^ Hz to 1 Hz and the oscillating voltage of 5 mV. The ionic conductivity (σ) was calculated by the following Eq. (3):

3 $${\rm{\sigma }}=\frac{l}{RA}$$

where l and A are the thickness and effective area of a membrane, respectively. And R is the impedance (Ω) of the membrane measured by EIS.

The lithium ion transference number (t^+^) was measured by sandwiching the membrane between two lithium electrodes. The lithium ion transference number (t^+^) was calculated by the following Eq. (4):

4 $${t}^{+}=\frac{{I}_{s}(\Delta V-{I}_{0}{R}_{0})}{{I}_{0}(\Delta V-{I}_{s}{R}_{s})}$$

where ΔV is the set polarization voltage, I_0_ and I_S_ are the initial and steady-state currents, and R_0_ and R_S_ are the initial and steady-state resistances of the passivating layers on the Li electrode.

## Supplementary information

 Supplementary Information
