# Supplementary material for: A facile non-solvent induced phase separation process for preparation of highly porous polybenzimidazole separator for lithium metal battery application
Source: Sci Rep. 2019 Dec 17;9:19320. doi: 10.1038/s41598-019-55865-6 (PMC6917766; doi:10.1038/s41598-019-55865-6)
Supplement: Supplementary file 1 — Supplementary Information [file 41598_2019_55865_MOESM1_ESM.docx]

**A facile non-solvent induced phase separation process for preparation of highly porous polybenzimidazole separator for lithium metal battery application**

Jiaying Wang^1^, Yang He^1^, Quan Wu^1^, Yunfeng Zhang*^1^, Zhiyuan Li^4^, Zhihong Liu*^2^, Shikang Huo^1^, Jiamig Dong^1^, Danli Zeng^1^, and Hansong Cheng^1^

^1^Sustainable Energy Laboratory, Faculty of Material Science and Chemistry, China University of Geosciences (Wuhan), 388 Lumo RD, Wuhan 430074, China. E-mail: zhangyf329@gmail.com

^2^School of Environment and Civil Engineering, Dongguan University of Technology, No.1, Daxue Road, Songshan Lake, Dongguan, Guangdong Province, 523808 P. R. China. E-mail: liuzhihong4523@163.com

^3^National Quality Supervision & Inspection Center of Lithium Battery Products (Shandong), Intelligent Manufacturing Town, Fuyuan 3rd Road, National High-tech Zone, Zaozhuang, Shandong, 277800, P. R. China


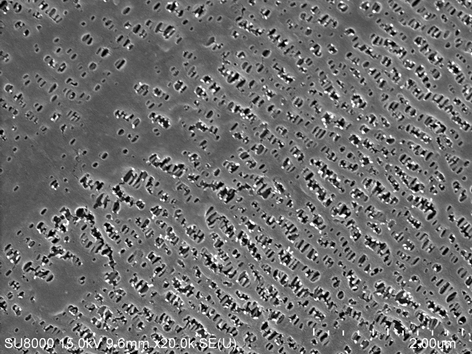


Fig. S1. The SEM image of the commercial PP separator.


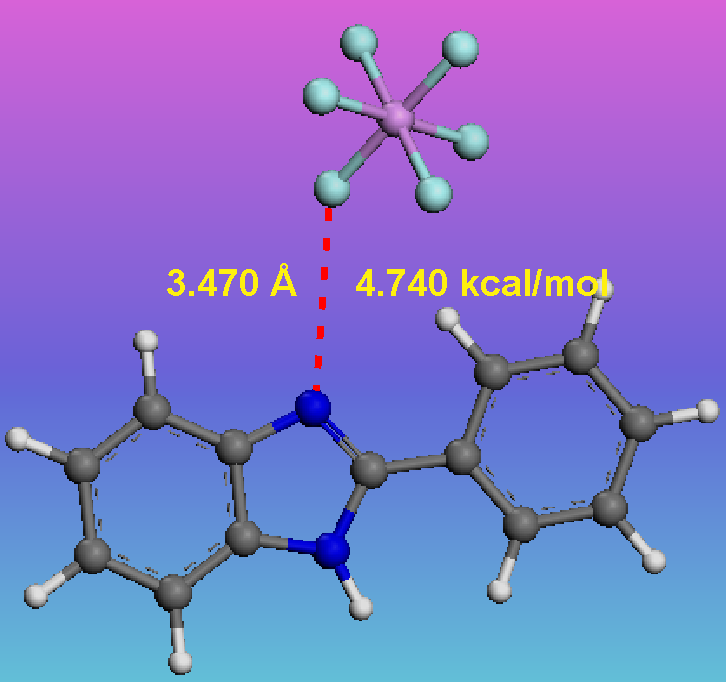


Fig.S2. the calculated bonding distance and bonding energy between PF_6_^-^ and electron-rich imidazole ring of PBI.

**Table S1. The calculated bonding distance and bonding energy of the PBI/LiPF_6_ electrolyte.**

| **bond** | **Bonding distance (**Å**)** | **Bonding energy (kcal/mol)** |
| --- | --- | --- |
| **Li^+^-PF_6_^-^** | 1.872 | 135.40 |
| **Li^+^---sp^2^ N of imidazole ring** | 1.988 | -49.194 |
| **PF_6_^-^--- sp^2^ N of imidazole ring** | 3.470 | 4.740 |


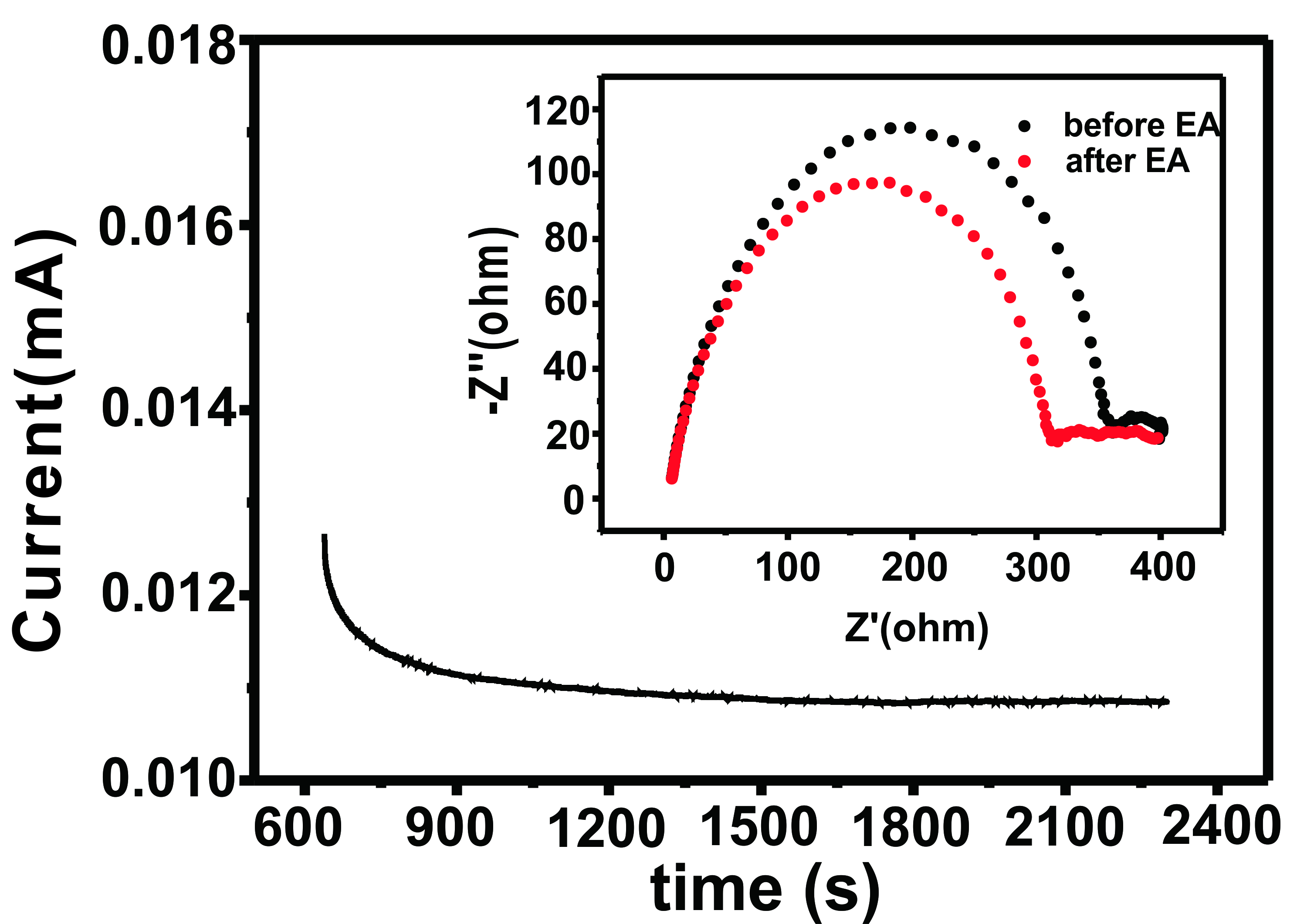


Fig. S3. The time-dependence response of dc polarization (inserted impedance spectra) for the PP/LiPF_6_ on the Li metal| PP/LiPF_6_|Li metal symmetric cell.


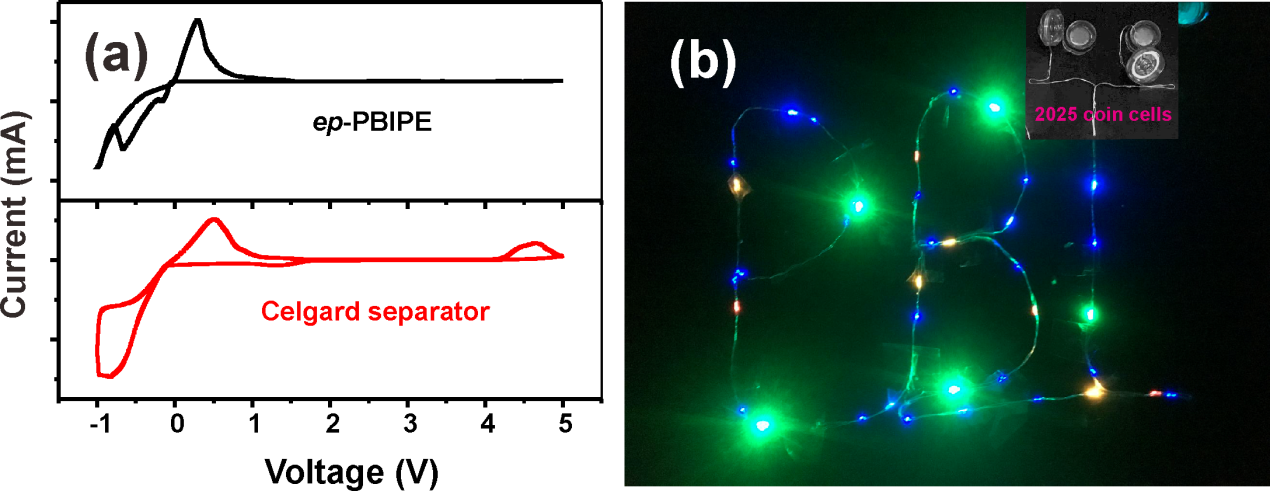


Fig. S4. Electrochemical stability of *ep*-PBIPE and Celgard separator with 1.0 M LiPF_6_ in EC/DMC electrolyte.
